# Early markers of adult obesity: a review

**DOI:** 10.1111/j.1467-789X.2011.00965.x

**Published:** 2012-12-16

**Authors:** T D Brisbois, A P Farmer, L J McCargar

**Affiliations:** 1 Department of Agricultural, Food and Nutritional Science, University of AlbertaEdmonton, Alberta, Canada; 2 Alberta Institute for Human Nutrition, University of AlbertaEdmonton, Alberta, Canada; 3 School of Public Health, University of AlbertaEdmonton, Alberta, Canada

**Keywords:** Adult obesity, child development, early childhood

## Abstract

The purpose of this review was to evaluate factors in early childhood (≤5 years of age) that are the most significant predictors of the development of obesity in adulthood. Factors of interest included exposures/insults in the prenatal period, infancy and early childhood, as well as other socio-demographic variables such as socioeconomic status (SES) or birth place that could impact all three time periods. An extensive electronic and systematic search initially resulted in 8,880 citations, after duplicates were removed. Specific inclusion and exclusion criteria were set, and following two screening processes, 135 studies were retained for detailed abstraction and analysis. A total of 42 variables were associated with obesity in adulthood; however, of these, only seven variables may be considered as potential early markers of obesity based on the reported associations. *Possible* early markers of obesity included maternal smoking and maternal weight gain during pregnancy. *Probable* early markers of obesity included maternal body mass index, childhood growth patterns (early rapid growth and early adiposity rebound), childhood obesity and father's employment (a proxy measure for SES in many studies). Health promotion programmes/agencies should consider these factors as reasonable targets to reduce the risk of adult obesity.

## Introduction

Obesity is considered to be a worldwide epidemic with little evidence that its incidence is declining or that it has even reached a plateau. In recent years, a large volume of research has focused on identifying early determinants or ‘warning signs’ of future development of obesity. Based on recent evidence, early-life experiences *in utero* and post-natal influences may induce permanent changes in physiologic function that programme the long-term regulation of energy balance. This subsequently may adversely impact obesity risk in later life.

Given that obesity may be programmed *in utero* and during early infancy, preventive measures should be initiated preconception, during pregnancy and continue throughout early childhood. However, it is not known which early markers are most important, nor is it known the relative contribution of individual early markers during critical periods in the obesity trajectory. Thus, the purpose of this review was to assess the literature to determine all potential prenatal, infant, childhood and socio-demographic markers (in children 0–5 years of age) which may have an impact on adult obesity. Given the breadth and scope of the review, specific inclusion parameters were set by the review advisory committee. As such, included studies were required to have measured outcomes for the same individuals for at least two time points during their lifetime (childhood and adulthood) for purposes of comparison. One time point early in life was required to assess variables in childhood (≤5 years of age) and a second time point was required in early to mid-adulthood (≥18 and ≤50 years of age). The overall goal was to identify an evidence-based list of early markers of obesity that could potentially be targeted for future prevention strategies. ***The final papers retained for the review are references***1–135.

## Methods and results

**Figure 1:**
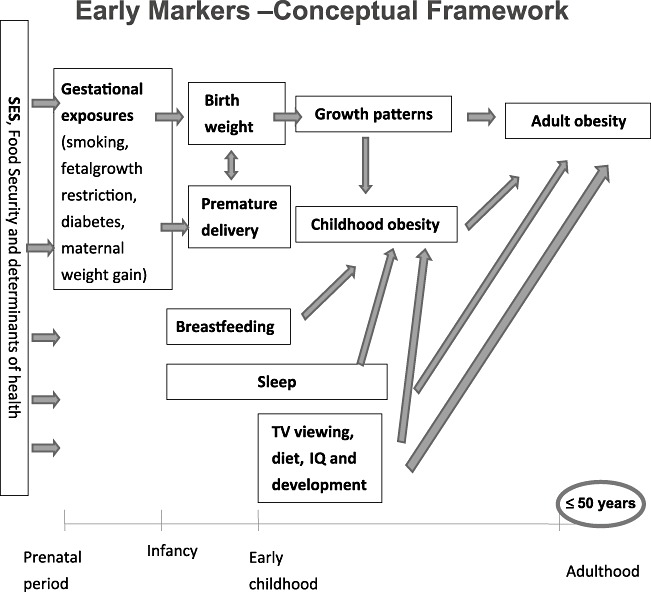
Conceptual framework for the review.

During initial planning of the review, a protocol and conceptual framework were developed (APF) to itemize previously identified factors associated with adult obesity (Fig. 1) and to guide the library search strategy and review process. These factors span three critical periods for children less than or equal to 5 years of age: prenatal (fetal development), infancy and early childhood. The review was not limited in scope to only biomarkers; and social determinants of health were also considered, including markers of socioeconomic status (SES) and food security. Gestational exposures such as smoking, fetal growth restriction, gestational diabetes and maternal weight gain for example were also considered. Furthermore, birth outcomes (birth weight, premature delivery), developmental characteristics (growth patterns, sleep patterns, IQ and cognitive development, and childhood obesity) and behaviours (breastfeeding, diet and television viewing) were all part of the framework.

### Literature search strategy

A review protocol developed by one of the investigators (APF) served as a blueprint for the literature search strategy. In December 2009, a medical librarian (DS–see Acknowledgements) developed search strategies specific to the different databases, which included the following: MEDLINE (OvidSP, 1950 to November Week 3, plus in-process citations up to Dec 22, 2009), EMBASE (OvidSP, 1980 to 2009 Week 50), EBMR Reviews (OvidSP – includes Cochrane Database of Systematic Reviews, DARE, Central Register of Controlled Trials, Cochrane Methodology Register, Health Technology Assessment, NHS Economic Evaluation, and ACP Journal Club), and ISI Web of Knowledge (includes Web of Science [1899–present], Biosis Previews [1926–present], CAB Abstracts [1910–present], and Food Science and Technology Abstracts [1969–present]). The search was inclusive of all languages. In addition to the electronic searches, hand searches of key nutrition and epidemiology journals (e.g. American Journal of Clinical Nutrition, Obesity Reviews, Journal of the American Dietetic Association, American Journal of Public Health) were also completed. Citations were managed and organized with a citation manager RefWorks©.

The initial search strategy was conducted in MEDLINE, then adapted to fit the indexing and search interface functionality of the other databases. For specific search terms for MEDLINE as an example, see Appendix A. The search strategy included relevant subject headings and text words for *obesity*, *overweight* and *adiposity* combined with terms related to developmental exposures, including but not limited to *breastfeeding, maternal smoking*, *gestational diabetes*, *nutrition*, *feeding, growth*, *sleep* and *socioeconomic status.* Broad terms related to early life and adulthood were also used in combination with *obesity* text words to retrieve additional results and ensure that the search was comprehensive. The citations list was reviewed by four research assistants and three researchers. The PRISMA (formerly known as QUORUM) protocol was followed for accounting of the screening process (136).

### Screening process and inclusion/exclusion criteria

The screening criteria were adapted based on the preliminary search results. At the outset of the review process, it was decided that only human research studies that included original research were to be included. In addition, only quantitative studies were included and qualitative studies (e.g. interviews, focus groups) and case reports were excluded. Genetic markers were not considered for this review; however, if other markers were also available in the same study, the citation was included. Markers of interest that were measured in children who were less than or equal to 5 years of age were included, and children and adolescents 6–17 years of age were excluded. For the adult measurements, only adults aged 18–50 years were included. A child and an adult measurement for the same individuals were required in all cases. Measurements of body mass index (BMI) or body composition (% body fat, waist circumference [WC] or waist-to-hip ratio [WHR]) were considered acceptable measures of adult body fat mass and obesity. Only healthy populations were included; studies were excluded if children had prediagnoses or existing health conditions, such as Down's syndrome, epilepsy, cancer or mental illness. The early marker data had to be measured in children who were 5 years of age or less. If, e.g. 5–9 year olds were investigated and followed into adulthood, this study would only be included if the data from the 5 year olds were presented separately. However, exceptions were made when the timing of parental data (e.g. education, employment and health status) were unspecified. These were assumed to be the same throughout the subject's childhood. All citations were reviewed independently by two reviewers. In cases where there was disagreement, the research team reviewed the studies and made decisions whether to include or exclude from the review.

### Data abstraction

All of the retained studies were abstracted independently by two research assistants. Discrepancies were resolved by a third research assistant or by team discussion. Detailed information related to the study characteristics was tabulated in an MSExcel (Microsoft Office, 2003) database. Specific study characteristics documented included citation information, document type, study type, definition of obesity, BMI data (e.i. measured or self-report), percentage body fat data (i.e. method of measurement, e.g. anthropometry, dual energy X-ray absorptiometry [DXA]), childhood marker of interest, sampling method, sample size, childhood and adult ages at time of measurement of key variables and proportion of males/females. Other study details documented included subject ethnicity, exclusion/inclusion criteria, indicators of bias such as percentage of subjects who completed the study or who were excluded from the study and sources of funding.

### Quality of the studies

Ranking the studies by specific quality assessments (e.g. criteria specific to study design and statistical analysis) was beyond the scope of this review; however, a number of factors were considered in evaluation of the results from each of the retained studies. Statistical rigor was carefully assessed, including the type of statistics completed and if there was adjustment for confounding variables. The type of study was also considered (prospective vs. retrospective) with the former being considered more rigorous. Similarly, measured variables vs. self-reported variables were considered more objective and reliable. It was preferred to have a measure of adult obesity (BMI > 30 or upper quintile of % body fat); however, some studies reported only overweight (BMI > 25) or trends in adult BMI/% fat mass.

Detailed data on the statistical analysis performed to describe the association between the early marker of obesity and adult obesity were tabulated. Given the diversity in the study designs and statistical analyses, a variety of statistics was reported. Information documented included risk assessment (odds ratio [OR], relative risk [RR] and hazard ratio [HR]), related means and frequencies (analysis of variance [anova], *t*-tests, chi-square), and associations (regressions, correlations). A *P* value of <0.05 was considered statistically significant. If trends were reported (often designated as *P* > 0.05 but *P* < 0.10), they were not abstracted. If multiple analyses were completed, the values adjusted for the most confounders were abstracted.

For the purposes of this paper, ‘possible’ and ‘probable’ early markers of adult obesity were identified. All identified markers were from studies that met all the predetermined criteria. A *possible* marker was defined as one whereby there were 6–10 studies that reported a positive relationship (in >80% of the studies) between the early marker and adult obesity in either female or male offspring. A *probable* marker was defined as one whereby there were at least 6–10 studies that reported a positive relationship (in 100% of the studies) between the early marker and adult obesity in either male or female offspring.

## Results

**Figure 2:**
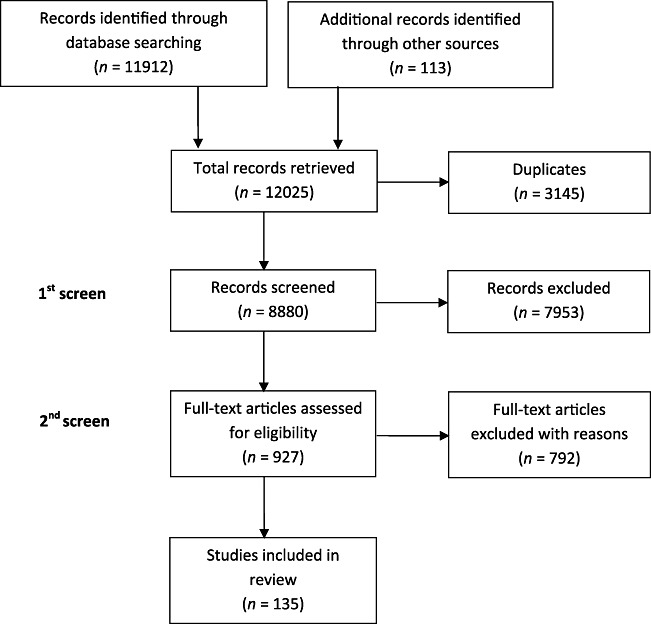
Literature flow to determine early markers of obesity. Adapted from The PRISMA Statement (136).

In total, 12,025 records were retrieved (Fig. 2). Duplicate references (3,145) were identified and removed. Of the remaining 8,880 studies, 7,953 were excluded and the remaining 927 studies were retrieved for full review in the second screening process, whereby all 927 papers were read independently by two research assistants. After detailed review of each study, the majority (*n* = 792) were excluded. After the second screen, 135 papers were retained for the final dataset (1–135). There was good agreement between reviewers for the first screen with a kappa value of 0.82, and for the second screen with a kappa value of 0.94. Where the full text article could not be found, an abstract of the same study information was retained for review. The majority of excluded studies (45%) in the second screening were due to an incorrect age for the outcomes of interest or childhood data were missing. Additional studies were excluded because they were research reviews (18%) or animal studies (11%). Due to time and resource constraints, we were unable to have 32 foreign language papers translated (including papers mainly from China, France, Italy, Germany and Spain). See Fig. 2 for flow diagram of the results of the entire screening process.

### Description of research articles retained for this review

#### Country of origin

The majority of included studies were carried out in European countries (45%, *n* = 61), followed by North America (26%, *n* = 35) and Australia/New Zealand (9%, *n* = 12). A smaller number of contributions came from Central America (7%, *n* = 9), South America (6%, *n* = 8), Asia (4%, *n* = 6) and the Middle East (3%, *n* = 4). Thus, the predominant results in this review represent developed countries, where obesity levels are high.

#### Study design and dates

Longitudinal prospective cohort studies that followed young children into adulthood comprised the majority of studies included in the review (*n* = 82, 61%). Seven studies had a start date in the 1920s and four studies began in the 1940s; with increased representation in the 1950s (*n* = 19), 1960s (*n* = 13), 1970s (*n* = 22) and 1980s (*n* = 12). The second study design was retrospective. These studies relied on the recall of adult participants to report on information from their early childhood (*n* = 53, 39%). Most of these studies were conducted more recently, from the 1970s (*n* = 5), 1980s (*n* = 7), 1990s (*n* = 16) and from the year 2000 onward (*n* = 20). Given there were data from prospective and retrospective studies, there is a mix of measured vs. self-reported early markers informing this review. Ten studies did not list the start date of their data collection.

### Evaluation of reported early markers of obesity

The original conceptual framework served as a guide for organizing this section into four subsections (Fig. 1): the prenatal period, infancy, early childhood and socio-demographic variables (which could impact all three time points). From the relevant studies, a total of 42 key variables/markers were identified. Markers to be discussed are listed at the beginning of each section. Those designated with an asterisk (*) are in addition to those identified in the initial framework. All markers were compared to adult BMI or an adult body fat measurement. Adult BMI was available for 124/135 papers and these results are presented herein. When BMI was not available (*n* = 11 papers), the results associated with the adult body fat measure were used and are identified throughout the review.

#### Prenatal period: 14 variables

Fourteen potential predictor variables were identified during the prenatal period. Many of these variables related to the mother during pregnancy, resulting in a direct influence on the developing fetus. These included maternal smoking, maternal diabetes, maternal weight gain, maternal weight, maternal height, maternal BMI, pre-eclampsia*, maternal dichlorodiphenyldichloroethylene (DDE) and polychlorinated biphenyl (PCB) levels*, and exposure to famine* during pregnancy. Paternal diabetes*, paternal weight*, paternal BMI*, both parents BMI* and intrauterine growth restriction (IUGR) were also identified as predictor variables.

##### Maternal BMI

Maternal BMI, maternal smoking and maternal weight gain during pregnancy were the most influential variables during the prenatal period. Maternal BMI emerged as a significant early marker for adult obesity of offspring in 17 studies; nine of these were prospective studies and the remaining were retrospective studies. Positive associations were shown in female offspring (7,53,97) and in both sexes (104,122,128,134). Increased risk or ORs were shown in the remaining studies (4,5,62,72,112,116,119,120,127) with one related to an adult adiposity measure (115). The variable ‘Maternal BMI’ was determined either through self-report, obtained from medical records or actual measurements of height and weight were completed. Of note, there was considerable variation across studies when ‘maternal BMI’ was assessed. Table 1 provides details about the studies related to maternal BMI and the development of obesity in their children in adulthood. The relationship held for different types of studies and different types of analyses.

**Table 1:** Mothers with a high body mass index and risk of their children developing adult obesity

Study (#, first author) (*n* = 17)	Type of study (P, R)	Country	Total sample *n* (%male)	Maternal BMI measure	Analysis/ results	± (f/m)*	*P* value	If data adjusted
(4) Al-Isa	R	Kuwait	842 (46%)	Participants reported mother obese	OR 1.88	+ (f)	<0.001	
					(CI – 1.28, 2.76)			
					Class 1 obesity			
					OR 2.71			
					(CI – 1.50, 4.90)			
					Class 2 obesity			
(5) Al-Isa	R	Kuwait	426 (0%)	Participants reported mother obese	Regression	+ (f)	<0.001	Adjusted
(7) Amine	R	United Arab Emirates	206 (0%)	Participants	Descriptive (Chi Sq)	+ (f)	<0.01	
				Reported mother obese				
(53) Karmaus	R	USA	151 (0%)	Mother's BMI	Correlation	+ (f)	NR	Adjusted
(62) Laitinen	P	Finland	6,280 (46%)	Mother's prepregnancy BMI	ancova	+ (f/m)	<0.001	Adjusted
(72) Magarey	P	Australia	155 (57%)	Mother obese (vs. normal wt)	RR 2.3	+ (f/m)	<0.001	Adjusted
(97) Ros	P	Sweden	624 (0%)	Mother's Prepregnancy BMI	Correlation	+ (f)	<0.001	Adjusted
(104) Schack- Nielsen	P	Denmark	1,660 (NR)	Mother's BMI	Correlation	+ (f/m)	NR	Adjusted
(112) Stettler	P	USA	300 (54%)	Mother's prepregnancy BMI	OR 1.20	+ (f/m)	<0.05	Adjusted
					(CI – 1.04, 1.39)			
(115) Stettler	P	USA	447 (51%)	Mother's prepregnancy body fat	OR 1.15	+ (f/m)	<0.001	Adjusted
					(CI – 1.06, 1.25) per unit increase in body fat†			
(116) Stuebe	R	USA	25,506 (0%)	Participants reported mother obese	OR 5.99† (CI – 4.69, 7.66)	+ (f)	NR	Adjusted
(119) Terry	P	USA	262 (0%)	Maternal prepregnancy BMI	Regression	+ (f)	<0.01	Adjusted
(120) Thomas	R	UK	9,019 (NR)	Maternal prepregnancy BMI	anova	+ (f/m)	<0.001	
(122) Verdy	R	Canada	734 (NR)	Mother's self- report adult BMI	Chi Sq	+ (fm)	<0.01	
(127) Whitaker	R	USA	854 (36%)	Maternal BMI >27.3 when child 1–5 years old	OR 3.6	+ (fm)	NR	Adjusted
					(CI – 2.1, 5.9)			
					when child 1–2 years old			
					OR 3.6			
					(CI – 2.2, 5.7)			
					when child 3–5 years old			
(128) Williams	P	New Zealand	925 (50%)	Mother's self- report (child 11 years)	Correlation	+ (f/m)	<0.05	
(134) Zadik	P	Israel	1,960 (49%)	Mother's BMI	Correlation	+ (f/m)	<0.001	

* (f/m) = females/males. Results are shown for adult female offspring (f), adult male offspring (m) or both (f/m).

† For this study (115), the early marker was evaluated with adult adiposity (not body mass index [BMI]), with excess body fat defined as >85th percentile for the sum of two skinfolds (triceps and subscapula).

† If mothers recalled prepregnancy, BMI was 29 (highest category specified).

anova, analysis of variance; CI, confidence interval; NR, not reported; OR, odds ratio; P, prospective study; R, retrospective study.

##### Maternal smoking

Smoking also emerged as a consistent and positive association, in that mothers' smoking increased the risk for the development of adult obesity in their children (data not shown). Seven studies addressed this issue (15,41,57,86,97,112,120). Maternal smoking showed a weak to moderate association with a higher adult BMI or higher rates of obesity in adulthood in six of the studies and no effect in one study (112). Of the six studies demonstrating a relationship, all but one had adjusted for covariates and four were prospective cohorts (15,41,86,97). Maternal smoking in general was based on a yes/no answer to smoking during pregnancy (15,57), after the fourth month of pregnancy (86,120) or whether the women had ever smoked (41,97); with one study not specifying the time frame (112). Strong correlations and moderate ORs were observed when comparing smokers vs. non-smokers. Maternal smoking appears to be a concern related to the development of adult obesity of the children.

##### Maternal weight gain

Maternal weight gain during pregnancy was addressed in six studies (38,74,104,115,116,119) (data not shown). Overall, the results were strong and consistent among the five studies that reported a BMI measurement in adulthood, in that a higher weight gain during pregnancy was positively associated with higher adult BMI, overweight or obesity of the offspring. This result remained for both types of study design and also for adult obesity development in both sexes. One study that used an adiposity outcome measure (skinfolds) in adulthood showed no association (115). Maternal weight gain has additional complexity because prepregnancy BMI must also be considered in terms of how much weight gain is considered appropriate (137). In one study, restrictive weight gain (<10 lb [4.5 kg]) was also associated with adult obesity (116); thus, the highest (>40 lb [18.2 kg]) and lowest weight gain categories were problematic, suggesting a U-shaped relationship. In another study (119), maternal weight gain predicted adult overweight in females at 20 years of age, but not at 40 years of age. Maternal weight gain during pregnancy appears to be a concern related to the development of adult obesity of the children.

#### Variables with mixed results or limited data

The variables listed herein either resulted in mixed and inconclusive results or there were very limited data available. These included ***IUGR***(15,67,114), ***maternal diabetes***(22,27,57,68), ***paternal diabetes***(27), ***maternal weight***(19,36,38,63), ***maternal height***(1,38,47,53,97), ***paternal weight***(19,36,63), ***paternal BMI***(4,5,7,72,127,128), ***pre-eclampsia***(97), ***maternal DDE and PCB levels***(53) and ***early malnutrition/famine exposure***(16,21,92,94,133).

A consistent but limited result was when ***both parents BMIs*** were high, there was an increased risk of overweight or obesity in their offspring in all studies (5,7,25,128,132), but one, where it was non-significant in males only (132). This was assessed through retrospective reports from adult offspring or measurements at various times during a prospective study of the children.

#### Summary of the prenatal period results

Fourteen variables (above) emerged in the literature review related to the prenatal period and adult BMI. IUGR, maternal diabetes, maternal weight and height, paternal weight and height, and early malnutrition/famine showed mixed results with no clear conclusion. Limited data were available (between 1 and 3 studies) for paternal diabetes, pre-eclampsia and maternal DDT/PCB levels. A consistent but limited result (*n* = 4 studies) was a positive association for both parents BMI and risk of adult obesity of their children. More consistent findings were observed for the relationships of *maternal BMI*, *maternal smoking* and *maternal weight gain* during pregnancy and adult BMI. Maternal smoking and maternal weight gain are *possible* early markers of adult obesity; and maternal BMI is a *probable* early marker of adult obesity.

### Infancy: 10 variables (* not in original framework)

Ten variables were investigated during the infancy period (defined as the first year of life). These included birth weight, differences between twins birth weights*, birth length*, ponderal index (PI), head circumference*, premature delivery, breastfeeding, infant hospital stay* and birth season*. In the initial search, breastfeeding was viewed as a yes/no variable. Complementary feeding was considered as a potential marker *a priori*; however, it did not appear as a measured variable in the final 135 papers that were reviewed.

#### Variables with mixed results or limited data

##### Birth weight

Birth weight was the most extensively studied variable (*n* = 55 studies) of all possible markers (6,9,14,17–19,26,28,30–33,37,38,41,44,47,48,50,52,53,56–59,61,64–67,69,71,76,80–82,84,90,92,97,98,100,103,110–112,114,115,117,119,121,122,126,129,132). This is likely due to the fact that there are usually reliable birth records within medical data systems that state weight and length of newborns. As well, mothers generally have good recall of the birth weights of their babies. Historically, birth weight has been considered as a key indicator of subsequent growth and development. Extremes in birth weight, both low and high, have been suggested as predisposing factors for the later development of obesity. ***Low birth weight*** (LBW) was evaluated in seven studies with mixed results and variable definitions of ‘low’ weight. Three studies (28,56,122) found no association between low birth weights and risk of adult obesity. Three studies reported an increased risk of obesity (44 [males only], (114)) or abdominal obesity (WHR) (61) with low birth weight; and two studies reported a decreased risk with LBW (47,53). Where definitions were provided, LBW or extremely LBW (28) included birth weight mean *Z* score of −0.90 (28), <2,600 g (56), <6 lb (122), lowest quintile of birth weight (114) <1,500 g (47), <2,500 g (53) and small for gestational age (SGA) <10th percentile (61).

When overall birth weight data were considered, a ***higher birth weight*** was positively associated with increased adult BMI or overweight and obesity rates in 25 studies, with no association observed in 18 studies. Of those studies showing positive results, 72% (16/25) were prospective cohort studies, 68% (17/25) were adjusted for covariates and 48% (12/25) were both. Of those studies reporting no association, 50% (9/18) were longitudinal, 67% (12/18) were adjusted for covariates and 33% (6/18) were both.

When birth weight was specifically evaluated with body fat measures, there was a positive association with WC in men (59), a negative association with WHR in women (59), a weak positive association with % body fat (measured by DXA) in both sexes (65) and no association in four other studies (66,67,115,126), using measures of body fat reference equations (66,67), skinfolds (115) and DXA (126). Overall, there was no clear trend in the birth weight data.

The variables listed herein either resulted in mixed and inconclusive results or there were very limited data available. These included ***differences between twins birth weights***(6,52,70), ***ponderal index***(1,14,23,24,31,58,67,77,78,90,100,114,121), ***head circumference***(14,114), ***neonatal hospital stay***(47) and ***birth season***(125).

##### Birth length

One of the most consistent findings was observed in the investigations of birth length. Of nine studies that assessed this variable, *no significant relationship* between birth length and adult BMI or overweight or obesity was reported in eight of them (14,23,52,57,71,97,100,110). A weak positive correlation was reported for males in the remaining study (121), and a weak positive correlation was reported in females only (not males) in one study (100). Birth length was not associated with adult % body fat in one study (66), nor in females only in another study (67).

##### Premature delivery

This variable also showed very consistent results. Nine of 10 studies reported *no association* between premature delivery and adult BMI, overweight or obesity (1,41,52,56,71,97,112,120). In one study, a weak negative correlation in males was reported between gestational age and adult overweight.

##### Breastfeeding

Breastfeeding was operationalized in a number of ways. It was an absolute yes/no variable or more commonly it was based on duration of breastfeeding, with subsequent categorization into monthly (or longer) intervals. Breastfeeding was investigated in nine studies, which also demonstrated quite consistent findings. Seven of the studies reported *no association* between breastfeeding and high adult BMI, overweight or obesity (9,19,60,79,83,91,102). Two studies reported a protective effect of breastfeeding; such that breastfed babies (>1 month duration) had a decreased prevalence of adult obesity in both males and females compared to those breastfed <1 month (123) or <1 month or not at all (99). Furthermore, babies who were breastfed longer (3–5 months duration), compared to those breastfed less than 1 month, had a decreased risk of adult obesity (123).

#### Summary of the infancy period results

Nine variables emerged in the literature review related to the infancy period and adult BMI. Birth weight (both low and high) and PI showed mixed results with no clear conclusion. Limited data were available (between 1 and 3 studies) for differential in twins' birth weight, head circumference, infant hospital stay and birth season. Birth length, premature delivery and breastfeeding showed consistent findings in that none of these variables were strongly associated with adult BMI, overweight or obesity. Overall, *no variables* in the infancy period appeared to have definitive potential to be considered as early markers of adult obesity.

### Early childhood: nine variables

Nine variables were investigated during the early childhood period (defined as ≥1 year of age and ≤5 years of age). These included rapid growth, adiposity rebound, childhood obesity, childhood height, sleep patterns, television viewing and behavioural problems. Childhood IQ and development, and a diet variable: sweet drink consumption were considered as potential markers *a priori*; however, these did not appear as measured variables in the final 135 papers that were reviewed.

#### Growth patterns: rapid growth/adiposity rebound

Rapid growth during infancy and early childhood and an early adiposity rebound (Table 2a,b**)** were two growth patterns that were strongly associated with the development of adult obesity. It has been suggested that these patterns may accelerate fat cell growth that is maintained for life and therefore may represent a ‘critical period’ influencing future body composition and fat deposition. Adiposity rebound is the physiological milestone whereby child growth (BMI) reaches a minimum level (usually between 4 and 8 years of age), and then BMI starts to increase again. The beginning of this second rise has come to be known as the adiposity rebound or BMI rebound (34,138). Different terminologies were used for these variables as well, including rapid growth, increased growth trajectory, increased growth velocity and earlier BMI rebound or adiposity rebound. The common factor of the 16 studies reviewed was that they represented periods of growth that were faster or at an earlier age than normal (or average). All studies, except one (65), were prospective cohort studies and all studies found some association between a rapid period of growth in early childhood and adult obesity. In some cases, it was during a particular period of time (i.e. 0–2 years of age); in some cases, it was only observed in one gender. Because of the variation in the time frames assessed, this is included in the early childhood section; however, growth patterns reported were between 0 and 5 years.

**2a:** Rapid early growth in childhood and risk of developing adult obesity

Study (#, first author) (*n* = 9)	Type of study (P, R)	Country	Total sample *n* (% male)	Rapid early growth	Analysis/results	± (f/m)*	*P* value	If data adjusted
(23) Corvalan	P	Guatemala	710 (50%)	BMI Δ	Regression	+ (f/m)	<0.05	Adjusted
				0–1 year		(f/m)	NSD	
				1–3 years		+ (f/m)	<0.01	
				3–7 years				
(37) Gasser	P	Europe	232 (52%)	wt gain	Correlation	+ (f/m)	<0.01	
				0–1 years				
(58) Kubo	P	Japan	244 (0%)	BMI Δ	Correlation	+ (f)	<0.05	
				0–1 years		(f)	NSD	
				1–5 years				
(65) Leunissen	R	Netherlands	312 (39%)	SGA and catch-up growth	ancova†	+ (f/m)	<0.05	Adjusted
(76) McCarthy	P	UK	679 (54%)	Growth velocity	Linear regression models			Adjusted
				0–5 months		+ (f/m)	<0.05	
				5 months–2 years		(f/m)	NSD	
						+ (f/m)	<0.001	
				2–5 years				
(109) Slining	P	Philippines	1,778 (NR)^d^	Rapid growth 0–2 years	Logistic regression	+ (f/m)	NR	Adjusted
(112) Stettler	P	USA	300 (54%)	Rapid growth 0–4 months	OR 6.72	+ (f/m)	<0.01	Adjusted
					(CI – 1.93, 23.4)			
					*both* adult BMI and body fat considered			
(113) Stettler	P	USA	653 (52%)	Early wt gain				Adjusted
				at 8 d	OR 1.28	+ (f/m)	<0.01	
					(CI – 1.08, 1.52)			
				at 112 d	OR 1.04	+ (f/m)	<0.01	
					(CI – 1.01, 1.08)			
(119) Terry	P	USA	261 (0%)	Rapid growth 1–7 years	Predictor of adult BMI @ 20 and 40 years	+ (f)		Adjusted

* (f/m) = females/males. Results are shown for adult female offspring (f), adult male offspring (m), or both (f/m).

† For this study (65), the early marker was evaluated with adult fat mass (not body mass index [BMI]), with body fat measured by dual energy X-ray absorptiometry.

Definitions: Δ, delta or change; ancova, analysis of covariance; CI, confidence interval, NR, not reported; NSD, no significant difference. No association; OR, odds ratio; P, prospective study; PAR, population attributable risk; R, retrospective study; SGA, small for gestational age.

**2b:** Adiposity rebound in childhood and risk of developing adult obesity

Study (#, first author) (*n* = 6)	Type of study (P, R)	Country	Total sample *n* (% male)	Adiposity rebound	Analysis/results	± (f/m)*	*P* value	If data adjusted
(31) Ezzahir	P	France	127 (49%)	Early adiposity rebound	anova	+ (f/m)	<0.05	Adjusted
(34) Freedman	P	USA	626 (44%)	Early adiposity rebound (<5 years)	Correlation	+ (f/m)	<0.001	Adjusted
(44) Guo	P	USA	338 (53%)	Early adiposity rebound	Regression	+ (f)	<0.001	Adjusted
					RR 2.27 (f)†	(m)	NSD	
(89) Prokopec	P	Czech Republic	158 (51%)	Early adiposity rebound (<5 years)	anova	+ (f/)	<0.05	
						+ (m)	<0.01	
(96) Rolland cachera	P	France	164 (52%)	Early (<5 years) vs. late adiposity rebound (>7 years)	anova	+ (f)	<0.01	
						+ (m)	<0.01	
(130) Williams	P	New Zealand	458 (52%)	Early adiposity rebound (<5.5 years) vs. (5.5–7.5 years)	RR 5.91 of obesity at 26 years (CI – 3.03, 11.55)	+ (f/m)		Adjusted

* (f/m) = females/males. Results are shown for adult female offspring (f), adult male offspring (m) or both (f/m).

† A girl with a body mass index (BMI) rebound 1 year earlier has 2.27 times the risk of having a BMI >25 as a woman than a girl with a later BMI rebound (44).

anova, analysis of variance; NSD, no significant difference. No association; P, prospective study; R, retrospective study.

Specifically, ***rapid infant/childhood growth*** (change in BMI) (see Table 2a) between 0 and 8 d of age (113), 0 and 4 months (112,113), 0 and 5 months (76), 0 and 1 year (23,37,58,119), 0 and 2 years (109), 2 and 5 years (76), 3 and 7 years (23) was associated with increased adult overweight or obesity. The association was observed in females only (58,119) or both sexes (23,37,76,109,112,113). Subjects who experienced catch-up growth (i.e. born SGA but achieving normal adult height) had a higher adult fat mass (measured by DXA) than controls (65). In a few studies (Table 2a), no association was shown between early rapid growth and obesity risk; however, there was no trend in these results that favoured a particular age or sex.

Many of the studies defined ‘***early adiposity rebound***’ as <5 years of age, and this was associated with increased risk or development of adult overweight or obesity ((34,44)[females only], (89,96,130)) (Table 2b). One study also reported that catch-up growth in infants who had been born small for gestational age had a greater influence on adult BMI if the age of ‘catch-up’ was later than 1 year of age (31).

#### Childhood obesity

A large number of studies investigated the presence of childhood obesity and high childhood BMI (*n* = 24) (Table 3). In almost all cases, there was a significant association between childhood obesity at <5 years of age and adult overweight or obesity. The majority of studies (22/24) were prospective cohort studies and the majority (21/24) had measured weight and height (and BMI) for the adult values. The age or age range of the children studied in this context was highly variable. Two studies reported no association with adult obesity based on specific childhood ages of 3 months and 1.5 years (76) and 1–2 years (127). The studies reporting positive associations/relationships assessed the children at 6 months (100), 1 year (89,103), 2 years (38,72), 3 years (46,103,128), 4 years (37,38) and 5 years of age (34,37,51,63,76,105,128). Some studies investigated age ranges of 1 month–5 years (58), 1–3 years (105), 1–4 years (55), 2–6 years (31), 3–5 years (127), ≥2 years (107) and ≥5 years (107). Four studies reported on childhood obesity in general (24,43,45,96). Overall, this group of studies investigated childhood BMI, the magnitude of change in BMI over a certain time frame, BMI percentile or specific cut-offs based on BMI percentile (>75th, >85th or >95th percentile representing overweight or obese).

**Table 3:** Childhood obesity and risk of developing adult obesity

Study (#, first author) (*n* = 24)	Type of study (P, R)	Country	Total sample *n* (% male)	Childhood obesity	Analysis/results	± (f/m)*	*P* value	If data adjusted
(24) Corvalan	P	Guatemala	1,559 (43%)	BMI at 5 years	Correlation	+ (f/m)	<0.05	
(31) Ezzahir	P	France	127 (49%)	BMI gain at 6 years	OR 1.9	+ (f/m)	<0.01	Adjusted
					(CI – 1.2, 3.0)			
(34) Freedman	P	USA	626 (44%)	BMI at 5 years	Correlation	+ (f/m)	<0.001	Adjusted
(35) Garn	P	USA	383 (44%)	Fatness (1–5 years)	RR 1.77† of adult fatness	+ (f/m)	<0.05	
(37) Gasser	P	Europe	232 (52%)	Early childhood BMI	RR increased	+ (f/m)	NR	
(38) Gigante	P	Brazil	2,250 (100%)	Wt for ht *Z* score at 2 + 4 years	anova	+ (m)	<0.001	Adjusted
(43) Guo	P	USA	459 (50%)	BMI percentile >75th in childhood†	RR of high BMI			
					(>25 kg m^−2^) at 18 years	+ (m)	NR	
					1.1–2.4	+ (f)	NR	
					1.3–3.1			
					RR of high BMI			
					(>25 kg m^−2^) at 30 years			
					1.1–1.4	+ (m)		
					1.2–1.8	+ (f)		
(45) Guo	P	USA	555 (50%)	Child BMI 95th vs. 50th percentile	OR 2.91	+ (f)	NR	N/A
					(CI – 1.34, 6.34)			
					OR 3.73	+ (m)	NR	
					(CI – 2.12,6.55)			
(46) Guo	P	USA	347 (48%)	BMI at age 3	anova	+ (f)	<0.05	
					obese vs. non-obese @ 35 years	(m)	NSD	
(49) Hawk	P	UK	621 (51%)	Body fat in childhood (skinfolds)	Correlation with adult fatness	+ (f/m)	NR	
(51) He	P	Sweden	3,650 (51%)	BMI at 5 years	RR 4.24 overwt at 18 years	+ (f/m)	NR	
(55) Kindblom	R	Sweden	612 (100%)	BMI 1–4 years	Correlation	+ (m)	<0.001	Adjusted
(58) Kubo	P	Japan	244 (0%)	BMI 3 months–5 years	Correlation	+ (f)	<0.001	
(63) Landhuis	P	New Zealand	972 (52%)	BMI at 5 years	OR 1.4	+ (f/m)	<0.001	Adjusted
					(CI – 1.18, 1.67)			
					Obesity at 32 years			
(72) Magarey	P	Australia	155 (57%)	BMI at 2 years	RR 2.72 Overwt at 20 years	+ (f/m)	NR	Adjusted
(76) McCarthy	P	UK	679 (54%)	BMI at 5 years	Correlation			Adjusted
					3 months, 1.5 years	(f/m)	NSD	
					5 years	+ (f/m)	<0.001	
(89) Prokopec	P	Czech republic	158 (51%)	BMI at 1 year (fat vs. lean)	RR 1.8	+ (f/m)		
					Obesity (>75th percentile) at 18 years		NR	
(96) Rolland-Cachera	P	France	102 (64%)	BMI >75th percentile	RR 2.0 Obesity (>75th percentile) at 20 years	+ (f/m)	NR	
(100) Sachdev	P	India	1,526 (58%)	Child BMI at 6 months	Correlation	+ (f/m)	NR	Adjusted
(103) Schack-Nielsen	P	Denmark	4,306 (NR)	BMI at 1 + 3 years	Correlation			Adjusted
					1 year	+ (f/m)	<0.05	
					3 years	+ (f/m)	<0.05	
(105) Schroeder	P	Guatemala	866 (58%)	BMI 1–5 years	Correlation			
					1–3 years	+ (f/m)		
					5 years	+ (f/m)		
(107) Siervogel	P	USA	459 (50%)	BMI ≥2 years§	Significant log OR with high adult BMI	+ (f)		
				BMI ≥5 years§	Significant log OR with high adult BMI	+ (m)		
(127) Whitaker	R	USA	854 (36%)	BMI >85th percentile	OR 1.3	(f/m)	NSD	Adjusted
					(CI – 0.7, 2.5)			
					1–2 years			
					OR 4.1	+ (f/m)		
					(CI – 2.5, 6.7)			
					3–5 years			
(128) Williams	P	New Zealand	925 (50%)	BMI 3 + 5 years	Correlation	+ (f/m)	<0.05	

* (f/m) = females/males. Results are shown for adult female offspring (f), adult male offspring (m) or both (f/m).

† For two studies (35,49), the early marker was evaluated with adult fat mass (not body mass index [BMI]), with body fat measured by skinfold measures.

† Early measures were completed ‘in childhood’. Specific ages were not provided.

§ A significant relationship (the log odds ratio) between childhood BMI and a high adult BMI was found in females beginning at 2 years of age and higher, and in males beginning at 5 years of age and higher.

CI, confidence interval; NSD, no significant difference. No association; NR, not reported; OR, odds ratio; P, prospective study; R, retrospective study.

In the few studies that showed no association, there was a tendency for the earlier ages to not be predictive of later obesity. For example, BMI at age 3 months and 1.5 years was not associated with adult BMI, but BMI at 5 years was associated with adult BMI (76). As well, 1- to 2-year-old children >85th BMI percentile did not have an increased risk of adult obesity; however, 3- to 5-year-old children (>85th BMI % percentile) did have an increased risk (127). Overall, childhood obesity appears to track into adulthood, especially from 2 years of age and older.

Two additional studies (Table 3) investigated the association between childhood body fatness and adult body fatness (35,49). One study demonstrated that 26.5% of initially obese (based on skinfold measures) children (1–5 years) were still obese as young adults (risk ratio 1.77) (35); the second study showed a moderate association between childhood and adulthood body fatness (also determined by skinfold measures) (49).

#### Variables with mixed results or limited data

The variables listed herein either resulted in mixed and inconclusive results or there were very limited data available. These included ***childhood height***(23,34,38,106), ***sleep patterns***(3,63,73), ***TV viewing***(124) and ***behavioural problems***(75).

#### Summary of the childhood period results

Seven variables emerged in the literature review related to early childhood; however, there was minimal evidence for four of them – childhood height (four studies with mixed results) sleep patterns (three papers), TV viewing (one paper) and behavioural problems (one paper). Thus, no conclusions can be made for these variables at this time. Strong, consistent findings were observed for *childhood growth patterns* (early rapid growth and early adiposity rebound) and *childhood obesity*. These variables are *probable* early markers of adult obesity.

### Socio-demographic factors: 13 variables (* not in original framework)

SES has long been considered as a factor related to the development of obesity. Many factors are studied as a proxy for SES, including income, education, employment or a composite of all three. Specific-related variables described herein include total SES (composite), household income, father's education, mother's education, parent's education, father's employment and parent (breadwinner) employment*. Mother's employment was considered as a potential marker; however, it did not appear as a measured variable in the final 135 papers that were reviewed. It was incorporated in a variable called ‘breadwinner employment’ where this represented either parent. Other demographic and familial characteristics including ethnicity*, birth place*, birth order*, number of siblings* and single-parent families* were also investigated.

Historically, excess weight was considered a sign of affluence; however, in more recent times, particularly in developed countries, low SES, rather than high SES, is more likely to be associated with increased obesity prevalence. This construct is complicated, however, and in part it has been attributed to difficulties maintaining a healthy diet, regular physical activity and general health behaviours. Of all direct and indirect indicators of SES reviewed, father's employment status showed a strong and consistent relationship with increased risk of their children developing obesity (Table 4). In particular, father's employment that was considered to be lower ‘status’ such as ‘blue collar’, ‘unskilled’ and ‘manual’ jobs, typically was associated with increased risk.

**Table 4:** Father's employment status and risk of their children developing adult obesity

Study (#, first author) (*n* = 17)	Type of study (P, R)	Country	Total sample *n* (% male)	Father's employment	Analysis/results	± (f/m)*	*P* value	If data adjusted
(8) Baecke	R	Netherlands	3,857 (46%)	Blue collar vs. professional	Regression	+ (f/m)	<0.001	Adjusted
(10) Ball	R	Australia	8,756 (0%)	Blue collar vs. professional	Regression	+ (f)	<0.001	Adjusted
(18) Bua	R	Denmark	562 (100%)	Unskilled vs. professional†	Mann–Whitney obese vs. controls	+ (m)	<0.01	
(29) Dundas	P	Scotland	7,095 (48%)	Low level employment (SC III – V)	OR	+ (f/m)	NR	Adjusted
					SC IV 1.56			
					(CI – 1.14, 2.13)			
					SC V 1.31			
					(CI – 0.95, 1.79)			
(41) Goldani	P	Brazil	1,189 (100%)	Unskilled vs. professional†	Regression	+ (m)	<0.05	Adjusted
(48) Hardy	P	UK	2,659 (NR)	Manual vs. non-manual; when child was 4 years	Multilevel models	+ (f/m)	<0.01	Adjusted
(59) Kuh	P	UK	3,200 (50%)	Manual vs. non-manual;	Regression§ with WC; and WHR	(f)	NSD§	Adjusted
						+ (m)	<0.05	
(61) Laitinen	P	Finland	5,771 (49%)	Unskilled vs. skilled; @ child's birth ≥ 90th percentile	Chi Sq with WHR	+ (f)	<0.05	Adjusted
						(m)	NSD	
(62) Laitinen	P	Finland	6,280 (46%)	Low vs. high level employment	Chi Sq	+ (f)	<0.05	Adjusted
						(m)	NSD	
(82) Parker	P	England	1,142 (43%)	Unskilled vs. professional @ child's birth	Correlation	(f)	NSD	Adjusted
						+ (m)	<0.05	
(85) Power	P	Britain	8,459 (48%)	Unskilled vs. professional	OR 1.99	+ (f)		
					(CI – 1.46, 2.72)	(m)		
					OR 2.19			
					(CI – 1.51, 3.19)			
(87) Power	P	Britain	7,485 (51%)	Unskilled vs. professional for females @ 7 years for males @ birth	OR 1.31	+ (f)		Adjusted
					(CI – 1.16, 1.47)	(m)		
					OR 1.19			
					(CI – 1.06, 1.35)			
(88) Power	P	Britain	12,274 (50%)	Manual vs. non-manual when child was 7 years	% SDS	+ (f)	9.2 vs. 4.1	
					>1.5 @ 23 years	(m)	8.2 vs. 4.6	
(93) Ravelli	R	Netherlands	283,028 (100%)	Manual vs. non-manual	Chi Sq	+ (m)	<0.05	
(118) Teasdale	R	Denmark	2,015 (57%)	Unskilled vs. professional (scale 0–7)	Regression	+ (f/m)	<0.05	Adjusted
(120) Thomas	R	UK	9,019 (NR)	Unskilled vs. professional @ birth	Chi Sq	+ (f/m)	<0.001	
(131) Wright	R	England	514 (45%)	Manual vs. professional at birth	anova @ 50 years	+ (f/m)	<0.01	

* (f/m) = females/males. Results are shown for adult female offspring (f), adult male offspring (m) or both (f/m).

† Svalastoga prestige-based rating scale (154).

† Social class based on father's occupation using the International System of Classification of Occupations (ISCO–1977) modified by Bettiol *et al*. (155).

§ For two studies (59,61), the early marker was evaluated with adult fat distribution (not body mass index [BMI]), with central adiposity measured as waist circumference/waist-to-hip ratio (WHR) (59) and WHR (61).

anova, analysis of variance; CI, confidence interval; NR, not reported; NSD, no significant difference. No association; OR, odds ratio; P, prospective study; R, retrospective study; SC, Social Class (III –V being considered lower SC); SDS = (log BMI − mean log BMI) ÷ SD log BMI.

#### Father's employment

Father's employment was evaluated in 15 studies resulting in consistent findings. In all studies, there was an association between fathers having lower employment status and adult obesity in their offspring (one or both sexes) (8,10,13,18,29,41,48,62,82,85,87,88,93,118,120)). In two studies, there was no association in males only (62) and females only (82). Many of the studies considered this variable as an SES measurement.

In addition, two studies investigated father's employment as it related to body fatness of their adult children (59,61) (Table 4). A manual occupation compared to non-manual occupation of the father was associated with higher adult WC and WHR in men, but not women (59); in contrast, an unskilled vs. skilled occupation of the father was associated with higher adult WHR in women, but not men (61).

#### Variables with mixed results or limited data

The variables listed herein either resulted in mixed and inconclusive results or there were very limited dataavailable. These included ***socioeconomic status*** (measured as a composite index representing per capita household income, key assets and maternal education) (1) and also based on a lifestyle questionnaire (25); ***household income*** was assessed in a number of ways, including the sum of the monthly income of all family members, or composite scores based on variables such as access to food, education and health care (11,36,38,42,108,132,135); ***father's education***(10,13,36) and ***mother's education***(10,15,36,57,90,112,115) were also investigated. Three studies evaluated ***parent's education*** together, and low education was associated with increased adult weight in males (54) in females (101) in African-American (both sexes, (95)) and in Caucasian women (95). Additional variables with inconclusive results included ***parent (breadwinner) employment*** defined as the occupation of the head of the household, or the higher income of one parent or higher values on an occupation score (12,17,63,132), ***ethnicity***(20,42,47,95,101), ***birth place*** investigated in two ways: with comparison of children being born in one country compared to another (2,39,40,50) or comparing those born in rural vs. urban centres (54,90,118); ***birth order***(8,95,112,115), ***number of siblings***(4,5,8,90,93,98) and ***single-parent family***(90).

#### Summary of the socio-demographic results

Twelve variables emerged in the literature review related to socio-demographic results. Household income, mother's education, parent (breadwinner) employment, ethnicity, birth place (country comparisons), birth order and the number of siblings showed mixed results with no clear conclusion. Limited data were available (between one and three studies) for SES (composite), father's education, parent's education, birth place (urban vs. rural comparison) and single-parent family. One key variable did show strong, consistent findings and that was *father's employment*. This variable is a *probable* early marker of adult obesity.

### Analytical considerations

#### Adult BMI vs. adult body fat

As mentioned previously, adult BMI was available for 124 of the 135 papers. When no BMI was available, a body fat measure was used (*n* = 11 papers). However, a number of variables were analysed in relation to both adult BMI and an adult body fat measure (*n* = 57). Of these, the results were the same for 74% (42/57) of the variables regarding their association with adult BMI or body fat. There were some differences in results between the remaining comparisons.

#### Childhood variable: measured vs. reported

For every paper, it was determined whether the childhood variable (e.g. maternal BMI, adiposity rebound) was measured or reported/self-reported or not specified. It is assumed that measured values would be more accurate; thus, results may have varied based on this factor. We reviewed the studies in Tables 1–4 and determined which studies had measured outcomes vs. reported/self-reported or not specified. In all but one table, the majority of the studies had measured variables: Table 1 (9/17), Table 2a (9/9), Table 2b (6/6), Table 3 (22/24) and Table 4 (8/17). In general, the reported results were in agreement with the measured results and would not affect the overall conclusions. Table 4, which reported on father's employment, would be expected to have more reported measures as some of the studies were retrospective accounts of an adult remembering their fathers' occupation when they were children. There was overall good agreement between the results of studies whether the childhood variables were measured or reported.

#### Adult outcome: measured vs. reported

For every paper, it was determined whether the adult outcome (e.g. BMI from height and weight; or body fat) was measured or reported/self-reported or not specified. It is assumed that measured values would be more accurate; thus, results may have varied based on this factor. We reviewed the studies in Tables 1–4 and determined which studies had measured outcomes vs. reported/self-reported or not specified. In all tables, the majority of the studies had measured variables: Table 1 (10/17), Table 2a (6/9), Table 2b (6/6), Table 3 (22/24) and Table 4 (11/17). There was overall good agreement between the results of studies whether the adult outcomes were measured or reported.

## Discussion

The purpose of this review was to identify an evidence-based list of early markers of obesity that could potentially be targeted for future prevention strategies. From the comprehensive review and evaluation of 42 variables in total, seven variables have consistent associations with the development of adult obesity. Maternal smoking and maternal weight gain in pregnancy showed consistent, but somewhat, limited results (*n* = 7/*n* = 6 studies, respectively). Maternal BMI, growth patterns during childhood (early rapid growth and early adiposity rebound), childhood obesity and father's employment emerged as the factors that were most consistently associated with or predictive of adult obesity of the offspring being studied.

Thus, preconception and prenatal health promotion programming for women of child-bearing age is highly warranted; with a focus on healthy weight, healthy weight gain during pregnancy and non-smoking. The post-natal time period is also a critical time for prevention of rapid weight gain in infants and children and prevention of the development of childhood obesity. The evidence in this review supports previous research regarding the tracking of childhood obesity through to adulthood. Low SES, which may limit access to health programmes, to healthy and safe play areas for children and to healthy food, is a societal issue that may play an important role in the development of obesity.

It is clear that these are not the only factors influencing obesity development. Forty-two variables that influence a child's life before the age of 5 years have been presented. Because each of the studies included in this review represent between 18 and 50 years of follow-up, influences vary somewhat depending on the decade, the country and the economic conditions of the time.

### Prenatal period

There are critical periods during fetal development that may influence adult body size (139). *In utero* programming refers to the capacity for fetal conditions to impact postnatal health outcomes (140). As such, maternal health can programme adipose tissue mass and distribution (140), as well as increase the risk of obesity and other metabolic disturbances in offspring (141). High maternal prepregnancy weight and adiposity and weight gain during pregnancy are important factors. The influence of a mother's weight status is likely a combination of genetic, biological, environmental and behavioural factors, all contributing to the impact of this variable in increasing the risk of obesity in their children. The intrauterine environment may work synergistically with genetic factors in metabolic programming (116) and a high maternal BMI in pregnancy has been associated with more rapid childhood growth (142). Pregnancy-specific weight gain has a persistent long-term impact on the offspring's adult BMI (119). Maternal smoking is also an important risk factor for childhood obesity and its persistence into adulthood (86,143). The mechanisms are unclear, although smoking may slow prenatal growth, resulting in postnatal catch-up growth (143) or it may be a marker of unhealthy lifestyle behaviours (143,144). Other researchers suggest that appetite regulation centres in the developing fetal brain may be affected by maternal smoking (145,146), or the effect of smoking may be mediated through food preferences and/or nutrient metabolism (147).

### Childhood

The growth acceleration theory proposed by Singhal and Lucas (148) suggests that it is accelerated weight change from birth (often called catch-up growth) that programmes a higher risk of metabolic abnormalities later in life. Rapid growth in infancy and subsequent obesity risk has been the subject of recent systematic reviews. Baird *et al*. (149) concluded that infants with the highest BMIs and/or those who grew more rapidly during the first 3 months to 2 years of life were more likely to be obese later in life. This association was consistent among a number of developed nations, across a range of ages and over the period of 1927–1994 (149). Other reviews demonstrated similar conclusions (150,151). The increased risk of obesity due to rapid early weight gain was independent of birth weight; however, infants with a longer period of weight gain may have a higher obesity risk (151). The present review determined that an early adiposity rebound is associated with a higher BMI in adulthood; however, this may have an impact even at younger ages. Researchers have also shown that an early adiposity rebound leads to a higher BMI in adolescence (89,138,152). Thus, it appears that obese infants and children have a high risk of becoming obese adolescents and subsequently obese adults. The prenatal and early childhood periods must be considered as key time points for prevention.

### Socio-demographic determinants

In an analysis of age, SES and obesity from the National Longitudinal Survey of Youth, Baum and Ruhm (153) determined that excess body weight in adulthood is inversely related to childhood SES. The authors suggest that this may be in part due to the fact that disadvantaged youth become disadvantaged adults, but the effect is also influenced by race/ethnicity (153). In the present review, father's employment was a consistent choice as a proxy for SES in much of the literature, and it was a strong indicator of later obesity in the offspring. Categorization of father's employment was often based on the degree of ‘prestige’ and years of education required for the type of employment (62), with the implication of higher financial reward. It is proposed that low SES in childhood leads to an accumulation of poor health behaviours throughout one's lifetime and the social inequalities associated with obesity (48).

### Strengths and limitations

This review provides a comprehensive assessment of the topic area, done using a systematic approach. It provides a synthesis of the extensive information available on early markers of obesity and contributes new knowledge. There are some limitations. Because of the large number of studies available on this topic, it was very labour-intensive to complete the detailed process of screening and abstracting. As it was *completed over several months*, some new personnel were required at later stages of the review to ensure its completion. Although the kappa values between researchers were good (>0.80), there were some discrepancies that had to be reconciled. As per research of this nature, the *variation in study design*, including methodology, sample size, years of follow-up, statistical analysis and outcome measurements, was problematic. Comparison between studies was often difficult and generalization of conclusions must be done with caution. A meta-analysis with detailed statistical evaluation was neither feasible nor appropriate given the diversity of primary outcome variables and study designs. The review is based on the *English-language* scientific literature only, which again may limit widespread generalization. Lastly, there was a *large range in the time frame* of the studies. The prospective studies were initiated as far back as the 1920s and the retrospective studies began in the 1970s. The obesity epidemic is a relatively recent phenomenon (last three decades); thus, the environmental determinants of obesity may have changed substantially over the last 90 years. However, the markers identified in this review are still relevant today and may just have been identified in a smaller subset of the population in earlier studies.

### Suggested future research

- Because of the ever-expanding knowledge in this area, a specific review focusing solely on each one of the seven possible or probable early markers of obesity reported herein is warranted. This review provides a scan of the studies where two measurements (childhood and adulthood) were available. An in-depth analysis of the seven markers identified would be valuable.
- Specific critical periods and/or cut-points could be determined for each variable for a risk assessment tool for early predictors of adult obesity.
- Identification of the mechanisms or social or environmental determinants of the significant relationships is required.
- Studies investigating the seven markers concurrently could determine how much of the variance in adult obesity is explained by these markers.
- Birth weight was extensively studied in the literature, more so than any other variable. Although no conclusions could be drawn at this time, it also warrants further consideration because of the significant literature showing positive associations between birth weight and adult obesity.

## Conclusions

Based on the literature review described earlier, and within the scope of the specific inclusion criteria, seven variables emerged as potential early markers of obesity: maternal smoking (before and during pregnancy) and maternal weight gain (during pregnancy) appear to be *possible* markers; maternal BMI, childhood growth patterns (early rapid growth and early adiposity rebound), childhood obesity and father's employment appear to be *probable* markers. Of note, the relationships described were associations with adult obesity and cannot be stated as causal in nature.

It is suggested by the evidence that early intervention is important. Modifiable risk factors have been identified that can be targeted to support healthy fetal growth and development that impact long-term outcomes in adulthood. Thus, in the *child-bearing years*, strategies for women to maintain a healthy weight, to avoid smoking and to gain a recommended amount of weight during pregnancy will have positive long-term health benefits for their offspring. During *infancy and childhood*, careful monitoring of height, weight and adiposity of children may be necessary to detect early changes in growth trajectories, and factors that may be influencing these changes. Lastly, programming that provides access to *vulnerable populations* is necessary because throughout a child's life, low SES is an influence on later development of adult obesity.

The authors responsibilities were as follows: TDB conducted the literature search and data collection, supervised research assistants and contributed to data interpretation and manuscript writing. APF developed the conceptual framework for the project, supervised the review and data collection process, and contributed to data interpretation and manuscript writing. LJM coordinated the project and contributed to data interpretation and manuscript organization and writing.

## Conflict of Interest Statement

The authors have no conflict of interest.

## Appendix

### Appendix A

#### MEDLINE (OvidSP) search terms

1950 – December 2009

1. Obesity/ep, et [Epidemiology, Etiology]
2. Overweight/ep, et [Epidemiology, Etiology]
3. Adiposity/
4. ([obes* or overweight or adipos* or body composition* or body mass] and [adult* or later or life or maternal or parent* or famil*]).ti.
5. (prenatal or neonat* or postnatal or fetus* or fetal or gestation* or development* or child* or infant* or infancy or early or offspring).mp.
6. (adult* or later or life or maternal or parent* or famil*).mp.
7. or/1–4
8. 5 and 6 and 7
9. ([prenatal or neonat* or postnatal or fetus* or fetal or gestation* or development* or child* or infant* or infancy or early or maternal or pregnan*] adj3 [expose* or exposure* or nutrition* or grow* or weight]).mp.
10. ([prenatal or neonat* or postnatal or fetus* or fetal or gestation* or child* or infant* or infancy or early] adj3 [develop* or programming]).mp.
11. 9 or 10
12. 8 and 11
13. ([prenatal or neonat* or postnatal or fetus* or fetal or gestation* or development* or child* or infant* or infancy or early or maternal or pregnan*] adj3 [expose* or exposure* or nutrition* or grow* or weight]).ti,sh.
14. ([prenatal or neonat* or postnatal or fetus* or fetal or gestation* or child* or infant* or infancy or early] adj3 [develop* or programming]).ti,sh.
15. (smoking and pregnan*).mp.
16. ([pregnan* or gestation*] adj3 diabet*).mp.
17. gestational age.mp.
18. (birth adj2 [weight or premature or pre term or preterm]).mp.
19. (bottle fe* or bottlefe* or breast fe* or breastfe* or complementary feeding).mp.
20. ([neonat* or postnatal or child* or infant* or infancy or early] adj3 [feed* or fed or diet*]).mp.
21. ([child* or infancy or infant*] and [sleep* or intelligence]).mp.
22. exp maternal behavior/ or exp parent-child relations/ or parenting/
23. Child Rearing/
24. parenting*.ti.
25. (adipos* adj2 rebound).mp.
26. socioeconomic factors/ or social class/
27. (socioeconomic* or socio economic).ti.
28. (food security or food insecurity).mp.
29. Food Supply/
30. Income/
31. Growth/ph [Physiology]
32. or/9–10,15–31
33. 8 and 32
34. *Obesity/ep, et [Epidemiology, Etiology]
35. 5 and 6 and 34
36. (cohort* or control* or longitudinal or follow-up or retrospective or prospective).mp.
37. risk.mp.
38. (associat* or predict*).mp. [mp = title, original title, abstract, name of substance word, subject heading word, unique identifier]
39. exp Age Factors/
40. or/36–39
41. 35 and 40
42. 33 or 41
